# Study of the Anti-Inflammatory Mechanism of β-Carotene Based on Network Pharmacology

**DOI:** 10.3390/molecules28227540

**Published:** 2023-11-11

**Authors:** Shilin Wu, Ran Chen, Jingyun Chen, Ning Yang, Kun Li, Zhen Zhang, Rongqing Zhang

**Affiliations:** 1 Zhejiang Provincial Key Laboratory of Applied Enzymology, Yangtze Delta Region Institute of Tsinghua University, Jiaxing 314006, China; mm18855193865@163.com (S.W.); cr17326264476@163.com (R.C.); chenjingyun0724@163.com (J.C.); anany0801@163.com (N.Y.); 18055462091@163.com (K.L.); 2 College of Fisheries and Life Science, Shanghai Ocean University, Shanghai 201306, China

**Keywords:** β-carotene, inflammatory, network pharmacology, molecular docking

## Abstract

β-carotene is known to have pharmacological effects such as anti-inflammatory, antioxidant, and anti-tumor properties. However, its main mechanism and related signaling pathways in the treatment of inflammation are still unclear. In this study, component target prediction was performed by using literature retrieval and the SwissTargetPrediction database. Disease targets were collected from various databases, including DisGeNET, OMIM, Drug Bank, and GeneCards. A protein–protein interaction (PPI) network was constructed, and enrichment analysis of gene ontology and biological pathways was carried out for important targets. The analysis showed that there were 191 unique targets of β-carotene after removing repeat sites. A total of 2067 targets from the three databases were integrated, 58 duplicate targets were removed, and 2009 potential disease action targets were obtained. Biological function enrichment analysis revealed 284 biological process (BP) entries, 31 cellular component (CC) entries, 55 molecular function (MF) entries, and 84 cellular pathways. The biological processes were mostly associated with various pathways and their regulation, whereas the cell components were mainly membrane components. The main molecular functions included RNA polymerase II transcription factor activity, DNA binding specific to the ligand activation sequence, DNA binding, steroid binding sequence-specific DNA binding, enzyme binding, and steroid hormone receptors. The pathways involved in the process included the TNF signaling pathway, sphingomyelin signaling pathway, and some disease pathways. Lastly, the anti-inflammatory signaling pathway of β-carotene was systematically analyzed using network pharmacology, while the molecular mechanism of β-carotene was further explored by molecular docking. In this study, the anti-inflammatory mechanism of β-carotene was preliminarily explored and predicted by bioinformatics methods, and further experiments will be designed to verify and confirm the predicted results, in order to finally reveal the anti-inflammatory mechanism of β-carotene.

## 1. Introduction

The inflammation response is a defense mechanism activated by the immune system when the body is exposed to harmful stimuli, such as microbial infection, allergens, radiation, etc. Cell surface pattern receptors identify harmful stimuli and activate corresponding signaling pathways. Immune cells then release pro-inflammatory cytokines that guide the synthesis of inflammatory mediators. This process leads to the activation and recruitment of white blood cells to the damaged sites for further treatment [1,2]. In the process of inflammation, a large number of inflammatory factors are secreted which transmit information within and between cells; this plays an important role in regulating inflammation [3]. Inflammation is a natural physiological reaction that can aid in the phagocytosis of pathogens, necrotic tissues, and other local reactions. It actively eliminates factors that induce damage and promotes the healing and recovery of injured tissues [4]. However, when the body’s ability to counteract injury is seriously disrupted by damage-inducing factors such as bacteria, viruses, UV rays, strong acids, and strong bases, the inflammatory response can intensify, leading to systemic symptoms such as parenchymal organ lesions, fever, and leukocytosis. In severe cases, systemic inflammatory response syndromes such as sepsis and toxemia may occur [5]. Autoimmune diseases can also cause the immune system to attack internal organs and tissues. Systemic lupus erythematosus (SLE) is an autoimmune disease in which the immune system mistakenly attacks its own tissue, causing inflammation and organ damage [6]. Infections or pro-inflammatory factors can also trigger immune reactions that cause tissue damage. For example, in viral myocarditis, virus invasion triggers an immune reaction that leads to myocardial cell damage and inflammation [7].

Traditional inflammatory treatment regimens include the use of steroidal anti-inflammatory drugs and nonsteroidal anti-inflammatory drugs. However, these drugs can cause various adverse reactions such as gastrointestinal diseases, cardiovascular and cerebrovascular diseases, infections, and even drug resistance [8]. Therefore, there is an urgent need to develop new drugs that are safe, efficient, have low toxicity, and are capable of reversing drug resistance.

In recent years, the study of natural products has gained increasing attention, particularly the study of β-carotene and its various physiological effects and functions that have been confirmed [9]. β-carotene is a natural carotenoid present in fruits and vegetables as well as being stored in algae, bacteria, and animals [10]. At present, about 750 kinds of natural carotenoids have been found, among which β-carotene is the most active typical representative and the best and most abundant vitamin A precursor in nature. it is also one of the most stable and common natural pigments in nature [11]. As an active source of vitamin A, it plays a significant role in maintaining visual function, promoting growth and development, regulating the immune response, and preserving mucosal integrity [12]. β-carotene has been shown to be a highly versatile molecule that interacts with multiple inflammatory molecular targets. Pharmacological studies in vitro and in vivo have also suggested that it may be a potential therapeutic agent for many inflammatory diseases. β-carotene is also an important signaling factor regulating tissue metabolism, with significant effects in regulating intestinal microbial flora, oxidative stress, and resisting inflammatory damage [13,14,15]. β-carotene can inhibit the production of NO, prostaglandin E2, and superoxide dismutase, while downregulating the expression of iNOS/cox-/NADPH oxidase proteins and mRNA, as well as inhibiting TNF-α to achieve the purpose of anti-inflammation [16]. Cui et al. [17] divided Wistar rats transplanted with liver tumor cells into a control group and groups with different concentrations of β-carotene. It was found that β-carotene can increase the number of NK cells in the blood of rats, and increase IL-2, TNF-α. The content of GSH-Px in the liver was increased, and the growth of tumor cells was inhibited, reducing the content of ALT and AST in the blood of mice with liver cancer. Li et al. [18] found that β-carotene decreased the oxidation level of porcine intestinal epithelial cells induced by LPS, significantly inhibited the expression of Caspase-3, and alleviated the inflammatory response induced by lipopolysaccharide by inhibiting NF-kB, JK2/STAT3 and JNK2/p38MAPK signaling pathways in macrophages. Furthermore, β-carotene possesses anti-inflammatory, antioxidant, and anti-tumor pharmacological effects [19,20,21].

However, the precise mechanism and related signaling pathways of β-carotene in treating inflammation remain unclear. In 2007, Hopkin [22] proposed the concept of network pharmacology, which combines drug action networks with biological networks to analyze drug–body interactions within specific nodes or modules in the network. This enables the understanding of the relationship between drugs and the body. Network pharmacology is an interdisciplinary and cutting-edge field that combines artificial intelligence and big data to study drugs in a systemic manner and is widely applied in drug compound discovery, mechanism of action explanation, drug combinations, and prescription compatibility law analysis [23]. Network pharmacology provides new technical support for rational drug use in clinical settings and the development of new drugs. Network pharmacology has the characteristics of integrity and of being systematic and comprehensive, which can better reveal the mechanism of drug action and guide the development of more drugs for treating various diseases [24]. Molecular docking is a method that uses chemoinformatics to simulate the geometric structure of molecules and intermolecular interactions to find the binding site of small molecular ligands and target protein molecules, achieving the purpose of forming a low-energy conformation after the two combines. This includes three interconnected parts: the recognition binding site, conformational search algorithm, and scoring function [25].

Alamri et al. [26] used network pharmacology and molecular docking technology to demonstrate that the active ingredient 5-Hydroxy-7,8 dimethoxyflavone in D. angustifolia can bind to AKT1, VEGFA, and EGFR, thereby participating in the body’s inflammatory response. Minjee et al. [27] used network pharmacology and molecular docking to identify the potential compounds and targets of *Fritillariae thunbergii* for treating influenza-related inflammation. Based on the analytical method of network pharmacology, this article aims to construct a β-carotene target network by assimilating and analyzing data. Consequently, this research enriches the biological functions and information regulation pathways of potential targets and analyzes anti-inflammatory genes, molecules, and signal pathways of β-carotene.

## 2. Results

### 2.1. Screening of β-Carotene Targets

According to SwissTargetPrediction and BATMAN-TCM prediction, 75 and 125 targets related to β-carotene were obtained, respectively. After removing the duplicate targets, a total of 191 β-carotene targets were identified.

### 2.2. Screening of Inflammatory Targets

Using “inflammation” as the keyword, relevant targets were searched for in the GeneCards, OMIM, TTD, and DisgeNET databases. A total of 1190 inflammation-related targets (score > 3) were retrieved from the GeneCards database. The score indicates the correlation between search results and search entries. The higher the score, the better the match between the search results and search terms. The OMIM database yielded 13 inflammatory targets and the TTD database yielded 158 targets. Finally, the DisgeNET database was searched for relevant targets, resulting in the identification of 467 targets. Integration of the results from all four databases yielded a total of 1828 targets, from which 354 repeats were removed, resulting in 1474 potential disease targets.

### 2.3. Screening of Anti-Inflammatory Candidate Targets of β-Carotene

**Figure 1:**
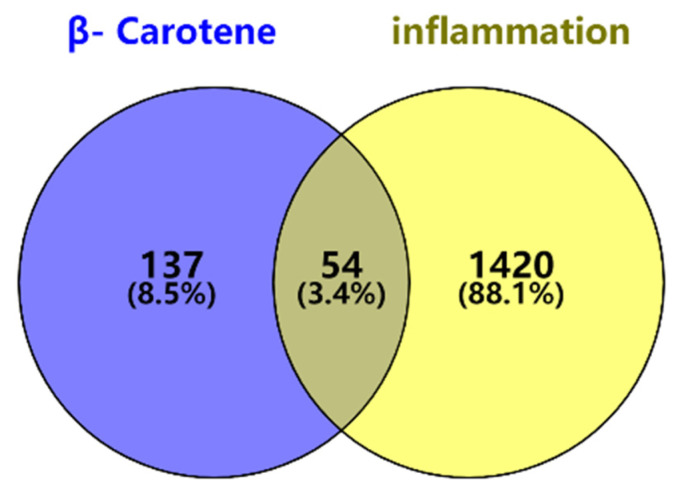
Venn diagram of β-carotene–inflammation effective targets.

A total of 54 candidate targets were identified through matching and mapping analysis of 1474 potential disease targets related to β-carotene. The results are illustrated in Figure 1 as a Venn diagram, where the light yellow portion on the right represents inflammatory targets, the light blue portion on the left represents β-carotene targets, the gray portion represents the intersection of the two, and the common targets of the two are highlighted.

### 2.4. Construction of the ‘Active Ingredient–Target Network

**Figure 2:**
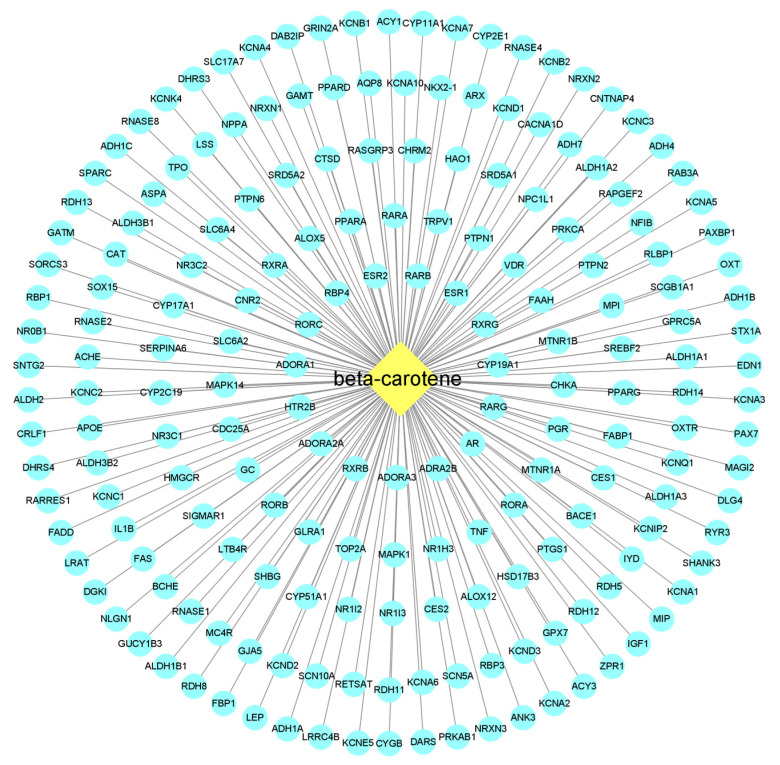
β-carotene-targets map. The middle diamond node represents the beta-carotene, and the surrounding circular nodes represent the targets that interact with the beta-carotene.

The active ingredient–action target network diagram of β-carotene and its corresponding 191 action targets was constructed by using Cytoscape 3.9.1 software (Figure 2). The part with a yellow background represents β-carotene, which is located in the center, and the part with a light cyan background represents the corresponding targets. According to the possible binding degree of the target molecule to β-carotene, all the targets are divided into five parts (the five rings in the figure). The closer to the center point, the greater the degree of possible binding to a β-carotene molecule.

### 2.5. Screening of Anti-Inflammatory Core Targets of β-Carotene and Construction of a PPI Network

**Figure 3:**
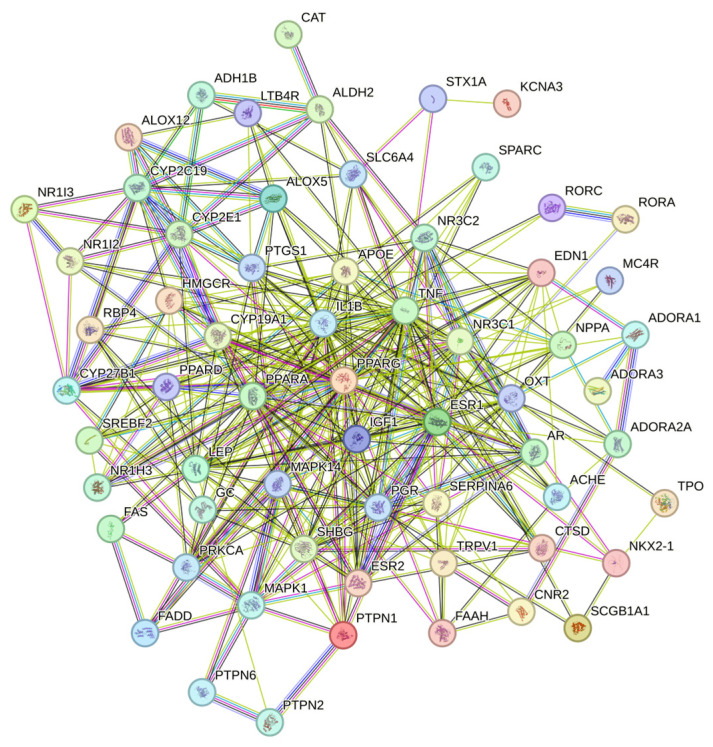
Candidate target protein interaction network. Nodes represent different targets, and the connections between nodes represent different interactions between them.

Protein–protein interaction refers to proteins that have physical contact with each other or functionally related proteins that form a network through complex and diverse interactions. Protein–protein interaction is the basis of exploring biological function, and the study of protein–protein interaction is of great significance for understanding and analyzing the biological function of drugs. Protein–protein interaction has been widely used in protein function prediction, disease research and prevention, drug candidate research, etc. Each node in Figure 3 represents a protein. Due to variable splicing and post-transcriptional modification in eukaryotes, a protein-coding gene may produce multiple proteins. Here, different proteins produced by the same gene are merged, and the letters marked on the node are the gene symbol of the corresponding gene. Some of the nodes in Figure 3 have spiral structures inside, which means that the three-dimensional structure of the protein is known, and if unknown, the interior of the nodes is empty. The connections between nodes represent the interactions between two proteins, and different colors correspond to different types of interactions, including known interactions, predicted interactions, and others [28].

**Figure 4:**
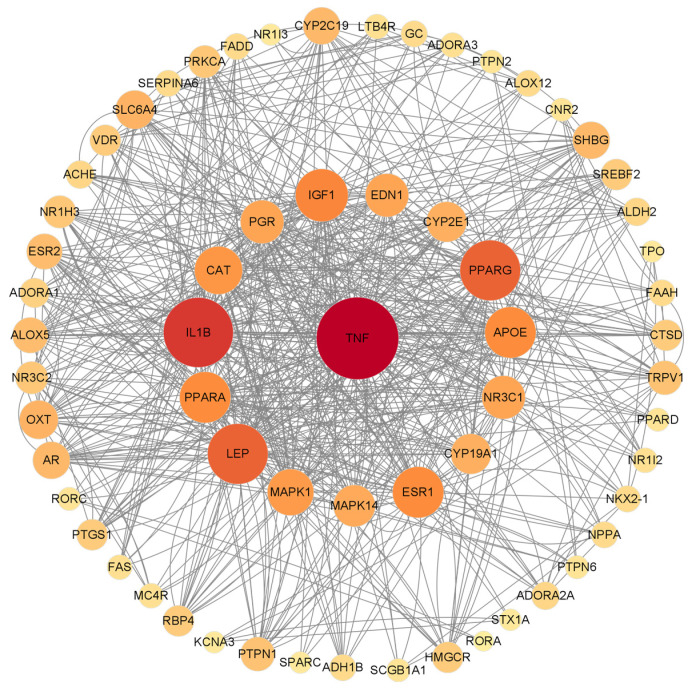
PPI network diagram of β-carotene-protein interaction of inflammation. The nodes are sorted by degree value. The larger the node, the darker the color, the higher the degree value, and vice versa. Nodes represent different targets, the connections between nodes represent the interaction between them, and the size of nodes represents the different possibility of binding with β-carotene.

Fifty-four potential targets were analyzed by using Cytoscape 3.9.1 software and a PPI network diagram was drawn (Figure 4). The ineffective targets were eliminated, and the key targets were screened out according to the degree value. The higher the degree value, the greater the possibility that the protein will play its role, and the more critical it is. Different color shades and node sizes were used to represent the difference of degree values. The larger the node, the darker the color, the larger the degree, and the more important the target. The analysis of the PPI network revealed a total of 54 nodes and 132 edges. Based on their degree values, the top ten targets were selected as TNF, IL1B, LEP, PPARG, IGF1, APOE, PPARA, ESR1, CAT, and MAPK1.

### 2.6. Functional Enrichment Analysis of the GO Gene and Enrichment Analysis of the KEGG Pathway

A bioinformatic analysis of 54 potential targets was carried out using the DAVID database with a screening condition of *p* < 0.05. A total of 370 entries were obtained via functional enrichment analysis, including 284 entries in BP (biological process), accounting for 76.76% of the results, 31 entries in CC (cellular component), accounting for 8.38%, and 55 entries in MF (molecular function), accounting for 14.86%.

**Figure 5:**
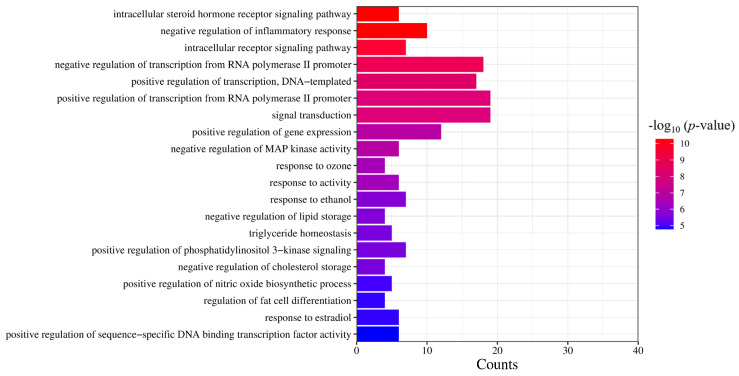
GO analysis of the biological process. The significance of the enrichment result is measured by the −log10 (*p*-value). The higher the −log10 (*p*-value), the more significant the enrichment result, and vice versa.

**Figure 6:**
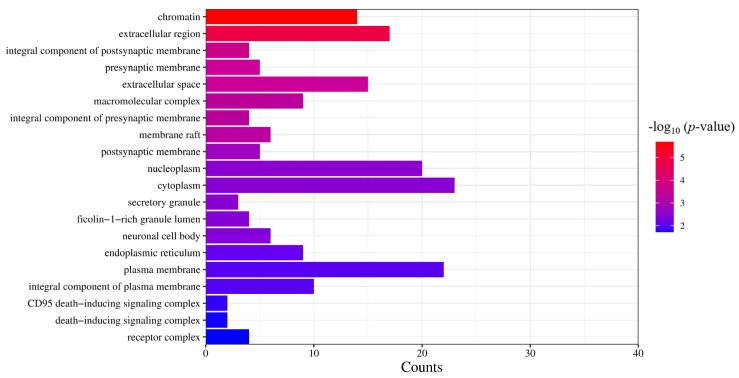
GO analysis of cell components. The significance of the enrichment result is measured by the −log10 (*p*-value). The higher the −log10 (*p*-value), the more significant the enrichment result, and vice versa.

**Figure 7:**
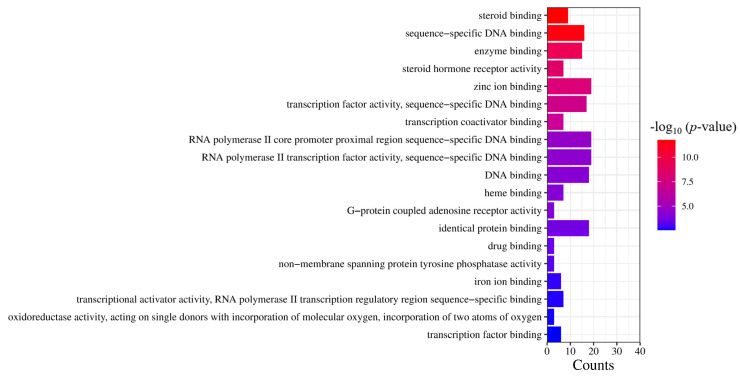
GO analysis of molecular function. The significance of the enrichment result is measured by the −log10 (*p*-value). The higher the −log10 (*p*-value), the more significant the enrichment result, and vice versa.

Based on the results of the molecular functional enrichment analysis presented in Table 1 and Figure 5, it appears that the potential targets for this study are mainly involved in the intracellular steroid hormone receptor signaling pathway, negative regulation of an inflammatory response, the intracellular receptor signaling pathway, negative regulation of transcription of the RNA polymerase II promoter, and negative transcription regulation of DNA templates. Additionally, cellular components identified in Table 2 and Figure 6 included chromatin, the extracellular region, an integral component of the postsynaptic membrane, extracellular space, and presynaptic membranes. Finally, molecular functions highlighted in Table 3 and Figure 7 included RNA polymerase II transcription factor activity, ligand-activated sequence-specific DNA binding, steroid binding, sequence-specific DNA binding, enzyme binding and steroid hormone receptor activity.

**Figure 8:**
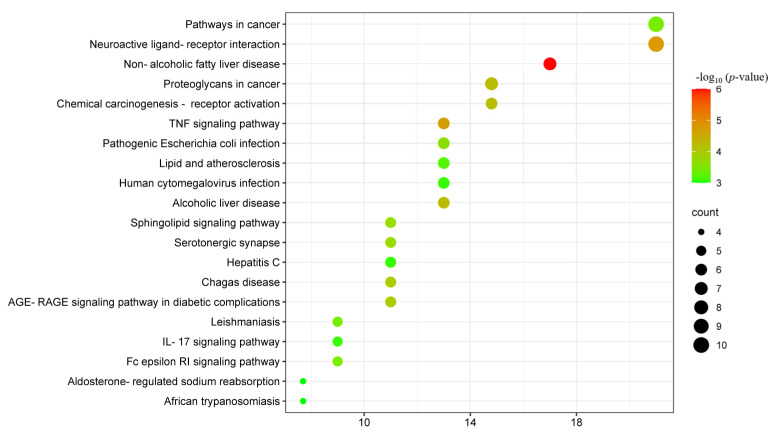
Bubble diagram of the KEGG pathway. The significance of the enrichment result is measured by the −log10 (*p*-value). The higher the −log10 (*p*-value), the more significant the enrichment result, and vice versa.

According to the genome-wide and metabolic pathway enrichment analysis of β-carotene anti-inflammatory targets, we have identified 84 pathways. As shown in Figure 8 and Table 4, among these paths, the top 20 paths were selected based on the number of targets and displayed using the Weishengxin platform. These pathways have been ranked in descending order according to −log10 (*p*-value). The top ten pathways are non-alcoholic fatty liver disease, neuroactive ligand–receptor interaction, TNF signaling pathway, proteoglycans in cancer, alcoholic liver disease, chemical carcinogenesis–receptor activation, AGE-RAGE signaling pathway in diabetic complications, Chagas disease, and sphingolipid signaling pathway.

### 2.7. Molecular Docking Results

**Figure 9:**
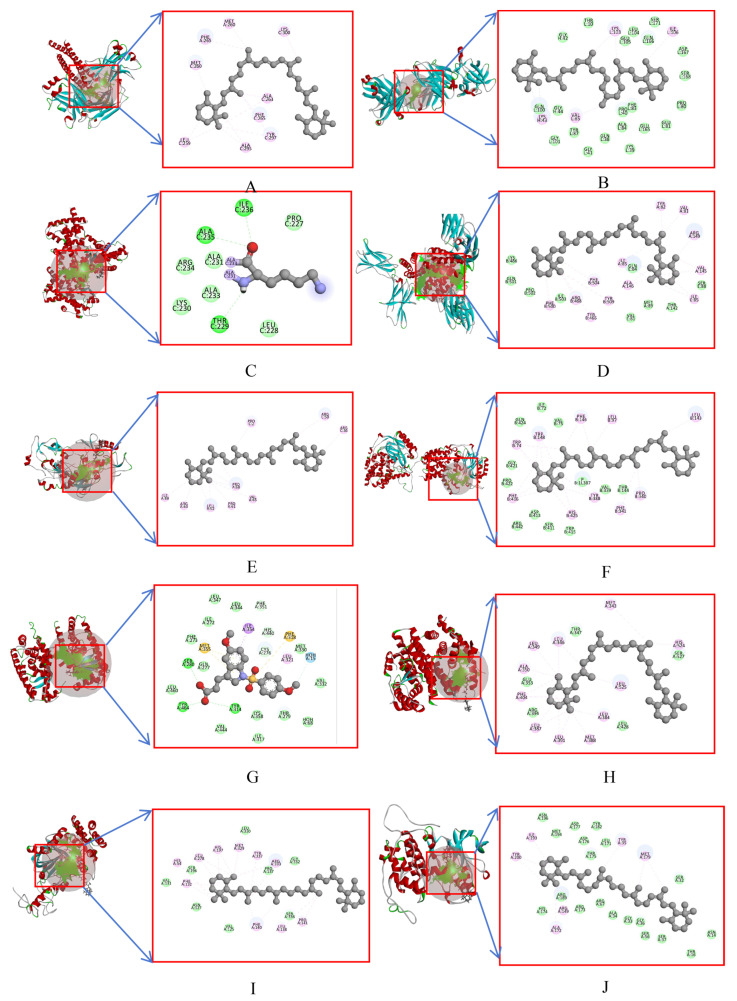
Docking pattern between β-carotene and the core target protein. (**A**) β-carotene–TNF, (**B**) β-carotene–IL1B, (**C**) β-carotene–PPARG, (**D**) β-carotene–LEP, (**E**) β-carotene–IGF1, (**F**) β-carotene–APOE, (**G**) β-carotene–PPARA, (**H**) β-carotene–ESR1, (**I**) β-carotene–CAT, (**J**) β-carotene–MAPK1; Cyan represents van der Waals, yellow for pi-sulfur, green for conventional hydrogen bond, gray for covalent bond, blue for halogen, and pink for pi-alkyl.

To score the molecular docking results, we utilized the LibDockScore function of the software. Based on the findings as illustrated in Figure 9, the results of β-carotene and the selected docking target are as follows: TNF with a score of 125.35, IL1B with a score of 113.32, PPARG with a score of 75.60, LEP with a score of 102.63, IGF1 with a score of 78.96, APOE with a score of 80.74, PPARA with a score of 78.96, ESR1 with a score of 99.96, CAT with a score of 103.31, and MAPK1 with a score of 97.67. Our results indicate that β-carotene has been successfully docked with the selected target molecules and, based on the score values obtained through the LibDockScore function, we can conclude that the binding effect between the molecules was efficient.

## 3. Discussion

The human body has a highly regulated system for controlling inflammation. This enables the effects of inflammation to be limited to specific times and places, resulting in a timely response to any harmful stimuli [29]. However, when this regulatory system breaks down or loses control, it can lead to a wide range of diseases, such as acute/chronic inflammatory, bowel disease, neurodegenerative diseases, and osteoarthritis, all of which can seriously impact overall health [30,31]. Inflammation is a primary feature of many pathological conditions, and it is a defense response that has evolved as part of the body’s continuous adaptive process. It is also the main pathological process that promotes the development of many diseases [32]. During the initial stages of an inflammatory response, the purpose of this response is to deal with tissue damage and invasive microorganisms, and to initiate the healing process [33]. The occurrence and development of inflammation are related to numerous signal transduction pathways, including the NF-kB pathway, MAPK pathway, etc. Specific blocking of these pathways can be achieved through up-regulation or down-regulation of related receptors in the pathway, slowing down the inflammatory response and enabling appropriate treatment of inflammation [34].

β-carotene is a natural pigment that has both coloring and nutritional functions and is permitted as a food additive. Research has shown that β-carotene possesses anti-inflammatory and anti-cancer properties and can improve animal immunity [35]. It is also capable of enhancing the quality of agricultural and livestock products [16]. Moreover, β-carotene can regulate lipid peroxidase related to immunity and inflammation, which plays important roles in protecting blood vessels, skin care, anti-tumor activity, enhancing immunity, and protecting the nervous system [36]. ROS (reactive oxygen species) is a term used to describe substances composed of oxygen, which are active in the natural environment or body. Although ROS perform essential roles in various life activities, excessive production can be harmful to the body. LPS (lipopolysaccharides) stimulate RAW264.7 macrophages to generate excessive ROS, which can be inhibited by β-carotene. The addition of β-carotene could enhance cell viability, inhibit the percentage content of ROS, reduce the secretion of inflammatory factors, and decrease the expression of the NF-kB p65 protein, which result in a positive effect on the treatment of inflammation [37]. Retinoic acid (RA) can complete the process of anti-inflammation by weakening the mRNA expression of TNF-α in microglia treated with LPS and inhibiting the release of TNF-α protein. Adding an appropriate amount of vitamin A to the diet can alter retinoic acid signal transduction (including the transcription factors involved in this pathway, such as ppara/pparag, etc.), and reduce the incidence of early urothelial cancer [38]. Yue et al. [39] found that vitamin A can regulate the proliferation and apoptosis of dermal cells under heat stress through IGF1 and Wnt10b signal transduction. Wang et al. [40] predicted the main targets of retinoic acid in the treatment of lipopolysaccharide-induced cardiac insufficiency to be PPARA, ITGAM, VCAM-1, IGF-1, and IL-6 through network pharmacology, and found that RA can reduce lipopolysaccharide-induced cardiac insufficiency by regulating the PI3K-Akt signaling pathway and key genes. In the course of analysis and study, it has been found that some of the targets identified in this study overlap with previously published inflammatory targets related to vitamin A or RA. This suggests that, as a precursor of vitamin A, β-carotene may express its anti-inflammatory effect through shared targets and pathways, at least to some extent.

In this study, 54 anti-inflammatory core targets of β-carotene were identified through network pharmacology. Among them, 10 key targets (TNF, IL1B, LEP, PPARG, IGF1, APOE, PPARA, ESR1, CAT, and MAPK1) were selected based on their degree value. Inflammatory factors are closely related to the occurrence of many diseases, and studies have shown that high expression of inflammatory cytokines is associated with drug resistance of cancer cells [41]. TNF (tumor necrosis factor) has typical cytokine characteristics and is a major inflammatory factor and pleiotropic cell regulatory protein. Excess local release of TNF can trigger an inflammatory response and immune response. The excessive presence of TNF-α and IL-β can activate vascular endothelial cells and neutrophils, playing an important role in the early stage of inflammation [42]. These inflammatory factors are the initiating factors of the cascade reaction and will further stimulate the release of other inflammatory factors, leading to a waterfall inflammatory response [43]. Moreover, studies have shown that cytokines such as Interferon-γ (IFN-γ) and IL-1B can induce macrophages to express a large amount of inducible nitric oxide synthase (iNOS) protein and produce NO, which functions as an immune molecule exerting anti-inflammatory and antiviral effects [44]. β-carotene can significantly inhibit LPS-induced release of IL-1β, IL-6 and TNF-α and down-regulate the expression of mRNA, and then inhibit NF-κB, JAK2/STAT3 and JNK/p38MAPK signal pathways in macrophages to alleviate LPS-induced inflammation [18]. PPARG can directly bind to p65, which is one of the members of NF-κB signal pathway. This binding can induce p65 to be degradated by proteasome and inhibit the expression of inflammation-related genes regulated by NF-κB [45]. PPAR is a member of the nuclear receptor superfamily, including PPARα, PPARβ/δ, and PPARg. After interaction with the ligand, PPAR then forms a heterodimer with retinoic acid X receptor, which plays a role in its target gene [46]. In addition, PPARα seems to inhibit its signaling pathway mainly through direct interaction with NF-kB, thereby reducing inflammation [47]. Leptin (Leptin, LEP) is a tumor necrosis factor family of cytokines, and is a polypeptide composed of 167 amino acids [48]. LEP is a regulatory molecule involved in the polycystic ovary syndrome (PCOS) cell model and acts as an upstream regulator of JAK1/STAT3-related inflammation and apoptosis in insulin-treated ovarian granulosa cells (OGCs). Up-regulation of LEP level is necessary to reduce apoptosis and inflammation by regulating the JAK1/STAT3 pathway [49]. LEP is a hormone/cytokine that participates in an inflammatory response by relying on the PI3K/mTOR cell signaling pathway [50]. Insulin-like growth factor 1 (IGF1) is a key regulator of cell proliferation, survival, differentiation, and metabolism. The role of IGF1 depends on the activation of the JAK2/STAT6 pathway rather than the typical RAS/Raf/ERK or PI3K/AKT pathway. Single cell sequencing showed that IGF1 could up-regulate the anti-inflammatory genes and upstream regulatory factors of most neutrophils and many macrophages after 3 days of treatment, down-regulating the pro-inflammatory gene and upstream regulatory factor at the same time [51]. Estrogen receptor alpha (ESR1) is one of the two intracellular receptors of estrogen, which is expressed by hepatocytes. ESR1 can inhibit liver regeneration by down-regulating the Wnt signal and decreasing the activity of cyclin D1 after chemical liver injury [52]. Apolipoprotein E (ApoE) is a major participant in cholesterol metabolism and reverse transport [53]. In a colitis model, APOE indirectly blocks the production of many inflammatory factors, including gTNF-α, KC, IL-17, and MIP-2, by preventing the nuclear accumulation of NF-kB and the activation of IKK [54]. The dysfunction of ESR1 will lead to neuroinflammation and further increase the risk of Alzheimer’s disease. Apolipoprotein E (APOE) can regulate the activity of ESR1 through CEBPB/ATF, mir-155-5p, and mir-1-3p [55]. Peroxisome proliferator-activated receptor (PPAR receptor) belongs to the nuclear receptor proteins, and is mainly involved in the regulation of transcription factor expression. Its target genes are mostly involved in lipid and glucose metabolism in high oxygen-consuming tissues such as the liver, muscle and heart [56]. Catenin (catenin, cats) is a cell signal transduction molecule and adhesion molecule that participates in intracellular signal transduction mediated by E-cadherin (E-cadherin) and plays an important role in cell adhesion, growth, proliferation and cell prognosis. Dong et al. [57] found that inhibition of the Wnt/β-Catenin signal pathway may be an effective way to regulate oxidative stress and inflammation in renal tissue. CAT can reduce the activation of NF-κ B, down-regulate the expression of immune-related cytokines, and then reduce the expression of inflammatory cytokines, so as to reduce the inflammatory response [58]. Mitogen-activated protein kinase (MAPK) is involved in signal transduction in physiological processes such as cell growth and apoptosis, and is activated by factors such as cytokines, neurotransmitters, cell stress, and cell adhesion [59]. Phosphorylation of JNK, ERK, p38, p65, and IκB can block the MAPK/NF-κB signal pathway and reduce the secretion of pro-inflammatory cytokines [60].

Functional enrichment analysis of the GO gene and the enrichment analysis of the KEGG pathway revealed that β-carotene can act on various proteins and participate in multiple pathways to achieve the effect of anti-inflammation.

## 4. Materials and Methods

### 4.1. Prediction of β-Carotene Targets

The molecular structure of β-carotene was retrieved using the PubChem database (https://pubchem.ncbi.nlm.nih.gov, accessed on 8 March 2023), and subsequently assessed through the SwissTargetPrediction (swisstargetprediction.ch, accessed on 8 March 2023) and BATMAN-TCM (http://bionet.ncpsb.org/batman-tcm/, accessed on 8 March 2023) online data sites to predict its potential targets [61]. In the BATMAN-TCM database, the parameter Target Prediction score cutoff value was set to 20, with an adjusted *p*-value of 0.05 utilized during target analysis [62].

### 4.2. Acquisition of Inflammatory Targets

To screen for relevant targets associated with “inflammation”, online network databases such as DisGeNET (https://www.disgenet.org/, accessed on 8 March 2023), GeneCards (https://www.genecards.org, accessed on 8 March 2023), TTD (Therapeutic Target Database, https://db.idrblab.net, accessed on 8 March 2023), and OMIM (https://www.omim.org, accessed on 8 March 2023) were utilized. These databases were employed to collate all targets, remove duplicates, and subsequently integrate the resulting targets.

### 4.3. Acquisition and Integration of Intersection Targets

The targets obtained for β-carotene and for inflammation were integrated and matched using the Weishengxin website (https://www.bioinformatics.com.cn, accessed on 8 March 2023) to obtain the common action targets of active substances and diseases, resulting in the identification of common targets. The intersection of these targets was represented using a Venn diagram for active substances and diseases. These intersection targets are considered as the anti-inflammatory targets of β-carotene.

### 4.4. Construction of the ‘Active Ingredient-Targets’ Network

The corresponding relationship between β-carotene and its target information was established using WPSOffice (12.1.0.15712) software, and saved as an Excel.xls file. This file was then imported into Cytoscape 3.9.1 software to construct the “active ingredient-targets” network.

### 4.5. Construction of Protein–Protein Interaction, PPI Network between Target Proteins of Anti-Inflammatory Action of β-Carotene

The collected common targets were imported into the STRING protein–protein interaction online database (https://string-db.org, accessed on 8 March 2023) with the human option selected. The minimum required interaction score was set to moderate confidence (0.400), and unrelated proteins were hidden before visualizing using the Cytoscape 3.9.1 software. The target result is sorted based on the degree value, and different colors and circle sizes are assigned to represent varying levels of gradient. This process resulted in the construction of a PPI network for the targets.

### 4.6. Functional Enrichment of the GO Gene and Enrichment of the KEGG Pathway

The common targets of β-carotene and inflammation were input into the DAVID database (https://david.ncifcrf.gov, accessed on 8 March 2023) for analysis of gene ontology (GO) function and Kyoto Encyclopedia of Gene and Genome (KEGG) pathways. GO enrichment was utilized to obtain possible cellular components (CC), molecular function (MF), and biological processes (BP) of the target genes involved in vivo. Through comprehensive analysis of these enriched targets, the biological functions of these genes were obtained. By enriching the signal pathways involved in the target through the KEGG pathway, the main anti-inflammatory signal pathways and biological processes of β-carotene were obtained [63]. Based on a *p*-value of less than 0.05, the top 20 GO enrichment analysis results and the top 20 KEGG enrichment results were selected for visualization and analysis [64]. The GO enrichment and the KEGG enrichment results were visually presented using the Weishengxin platform.

### 4.7. Molecular Docking

The RCSB database (https://www.rcsb.org/, accessed on 8 March 2023) was utilized to retrieve and download the structure of the core target proteins in PDB format. The 3D structure of β-carotene was obtained from the PubChem database and saved in SDF format. These structures were then prepared using Discovery Studio 2019 (v19.1.0.) software, which involved removal and hydrogenation of the molecular structure of the core target protein. Molecular docking was subsequently performed.

## 5. Conclusions

To conclude, this study applied network pharmacology to identify potential core targets and possible signal pathways of the anti-inflammatory effects of β-carotene. The active ingredient showed a good affinity with the targets, which were verified by molecular docking analysis. TNF, IL1B, and LEP were identified as key targets for the anti-inflammatory effects of β-carotene. These findings may contribute to the development of β-carotene and its potential use as a therapeutic agent for inflammation-related diseases.

## Figures and Tables

**Table 1:** Diagram of biological process information.

GO Serial Number	Description	−log10 (*p*-Value)
GO:0030518	intracellular steroid hormone receptor signaling pathway	10.28
GO:0050728	negative regulation of inflammatory response	10.15
GO:0030522	intracellular receptor signaling pathway	9.37
GO:0000122	negative regulation of transcription from RNA polymerase II promoter	8.55
GO:0045893	positive regulation of transcription, DNA-templated	8.35
GO:0045944	positive regulation of transcription from RNA polymerase II promoter	8.08
GO:0007165	signal transduction	7.77
GO:0010628	positive regulation of gene expression	6.93
GO:0043407	negative regulation of MAP kinase activity	6.89
GO:0010193	response to ozone	6.44
GO:0014823	response to activity	6.32
GO:0045471	response to ethanol	5.77
GO:0010888	negative regulation of lipid storage	5.66
GO:0070328	triglyceride homeostasis	5.59
GO:0014068	positive regulation of phosphatidylinositol 3-kinase signaling	5.54
GO:0010887	negative regulation of cholesterol storage	5.53
GO:0045429	positive regulation of nitric oxide biosynthetic process	5.05
GO:0045598	regulation of fat cell differentiation	4.92
GO:0032355	response to estradiol	4.90
GO:0051091	positive regulation of sequence-specific DNA binding transcription factor activity	4.78

The degree of significance of enrichment results is measured by the value of −log10 (*p*-value). The higher the value of −log10 (*p*-value), the greater the significance of the enrichment results.

**Table 2:** Information on cell components.

GO Serial Number	Description	−log10 (*p*-Value)
GO:0000785	chromatin	5.69
GO:0005576	extracellular region	4.85
GO:0099055	integral component of postsynaptic membrane	3.65
GO:0005615	extracellular space	3.53
GO:0042734	presynaptic membrane	3.53
GO:0032991	macromolecular complex	3.39
GO:0099056	integral component of presynaptic membrane	3.35
GO:0045121	membrane raft	3.32
GO:0045211	postsynaptic membrane	2.75
GO:0005654	nucleoplasm	2.47
GO:0030141	secretory granule	2.44
GO:0005737	cytoplasm	2.45
GO:1904813	ficolin-1-rich granule lumen	2.40
GO:0043025	neuronal cell body	2.39
GO:0005783	endoplasmic reticulum	2.07
GO:0005886	plasma membrane	2.04
GO:0005887	integral component of plasma membrane	2.00
GO:0031265	CD95 death-inducing signaling complex	1.81
GO:0031264	death-inducing signaling complex	1.75
GO:0043235	receptor complex	1.72

The level of significance of enrichment results is determined by the value of −log10 (*p*-value). The greater the value of −log10 (*p*-value), the higher the significance of the enrichment results.

**Table 3:** Molecular function information table.

GO Serial Number	Description	−log10 (*p*-Value)
GO:0004879	RNA polymerase II transcription factor activity, ligand-activated sequence-specific DNA binding	21.87
GO:0005496	steroid binding	11.76
GO:0043565	sequence-specific DNA binding	11.31
GO:0019899	enzyme binding	9.20
GO:0003707	steroid hormone receptor activity	8.34
GO:0008270	zinc ion binding	7.99
GO:0003700	transcription factor activity, sequence-specific DNA binding	7.49
GO:0001223	transcription coactivator binding	6.70
GO:0000978	RNA polymerase II core promoter proximal region sequence-specific DNA binding	4.68
GO:0000981	RNA polymerase II transcription factor activity, sequence-specific DNA binding	4.41
GO:0003677	DNA binding	4.22
GO:0020037	heme binding	4.18
GO:0001609	G-protein coupled adenosine receptor activity	4.12
GO:0042802	identical protein binding	3.69
GO:0008144	drug binding	3.38
GO:0004726	non-membrane spanning protein tyrosine phosphatase activity	3.30
GO:0005506	iron ion binding	2.89
GO:0001228	transcriptional activator activity, RNA polymerase II transcription regulatory region sequence-specific binding	2.67
GO:0016702	oxidoreductase activity, acting on single donors with incorporation of molecular oxygen, incorporation of two atoms of oxygen	2.62
GO:0008134	transcription factor binding	2.56

The level of significance of enrichment results is determined by the value of −log 10 (*p*-value). The greater the value of −log 10 (*p*-value), the higher the significance of the enrichment results.

**Table 4:** KEGG information.

KEGG Serial Number	Description	−log10 (*p*-Value)
hsa04932	non-alcoholic fatty liver disease	5.79
hsa04080	neuroactive ligand-receptor interaction	4.65
hsa04668	TNF signaling pathway	4.52
hsa05205	proteoglycans in cancer	3.95
hsa04936	alcoholic liver disease	3.95
hsa05207	chemical carcinogenesis—receptor activation	3.86
hsa04933	AGE-RAGE signaling pathway in diabetic complications	3.69
hsa05142	Chagas disease	3.65
hsa04726	serotonergic synapse	3.41
hsa04071	sphingolipid signaling pathway	3.34
hsa05200	pathways in cancer	3.32
hsa04664	Fc epsilon RI signaling pathway	3.30
hsa05130	pathogenic Escherichia coli infection	3.18
hsa05140	leishmaniasis	3.10
hsa05417	lipid and atherosclerosis	2.98
hsa05140	leishmaniasis	2.86
hsa05130	pathogenic Escherichia coli infection	2.84
hsa05200	pathways in cancer	2.80
hsa04960	aldosterone-regulated sodium reabsorption	2.80
hsa05417	lipid and atherosclerosis	2.65

The significance of the enrichment result is measured by the −log10 (*p*-value). The higher the −log10 (*p*-value), the more significant the enrichment result, and vice versa.

## Data Availability

The data presented in this manuscript are available from the corresponding author upon request.
